# Diseases of the Urinary Organs

**Published:** 1859-02

**Authors:** 


ART. VII. — *Diseases of the Urinary Organs. A Compendium of their Diagnosis, Pathology and Treatment.* By William Wallace Morland, M. D., Fellow of the Massachussetts Medical Society; Member of the Boston Society for Medical Improvement; one of the Attending Surgeons at the Central Office of the Boston Dispensary; Corresponding Member of the Glasgow Medico-Chirurgical Society. With Illustrations. Philadelphia: Blanchard and Lea. 1858.

This volume has been for some time on our table, through the politeness of the enterprising publisher, and has long since received quite a careful examination, though we have not before had time to put our thoughts and opinions of it on paper. A compendium of the diseases of the urinary organs has been a desideratum; especially one which gave equal attention to the medical and surgical views of the subject. We have some works, which embrace only certain classes of urinary diseases, which are very admirable. The well known work on urinary deposits by Bird, is certainly one of the most useful productions of late years, the value of which, every one who has attempted the microscopic investigation of the urine has experienced. We have also "Manuals of the Blood and Urine," by Griffith, Rees, and Marwick, and a little work on renal diseases by Frick. Neither of these, however, are as useful as the work of Dr. Bird. This part of the subject is not treated of by Dr. Morland, and it is well, perhaps, that he has omitted it; for though we are now able to recognize all the urinary crystals by means of the microscope, and we can tell, with almost unerring certainty, the exact composition of any urinary sediment; the relations of these deposits to disease are vague and uncertain, and it is only in a few cases that a knowledge of the urinary deposit is of much advantage to us in diagnosis, treatment, or prognosis.

The author has drawn largely upon the admirable treatise of Dr. Johnson on diseases of the kidney, and upon the Gross' work on the urinary organs. Both of them are excellent works; especially the work of Dr. Gross, with which every American surgeon is acquainted, and which is the most complete treatise extant upon the surgical diseases of the urinary organs. The section referring to diseases of the kidneys have been modeled after Johnson, and in the section devoted to surgical diseases, the author has closely followed Dr. Gross. The work is composed of the substance of two prize essays presented to the Boylston Medical Committee in 1855 and 1857. In regard to the arrangement of the subjects, we think that the author has made a great mistake, one which will very much impair the usefulness of the book; *i. e.*, its separation into two distinct parts; one treating of diagnosis simply, and the other of pathology and treatment. This makes it necessary to have two sections devoted to each disease; and when one has read what the author has to say in the first part, of diagnosis, he is compelled to refer to the second part, for the pathology and treatment. The matter is all there, it is true, but this arrangement will be a source of annoyance to readers who may have occasion to refer to the work for information on a particular disease. We experienced this annoyance when we first looked over the work: the connexion seemed to us entirely broken. When we read, for example, the account of Bright's disease, or, to take the nomenclature of Dr. Johnson, which is adopted by the author, "Fatty degeneration of the Kidney," we were surprised that so important a disease should have been treated of in a chapter of only ten pages; but nearly two hundred pages farther on, we have thirty pages devoted to the pathology and treatment of this disorde

In our humble judgement, it would have been infinitely better to have had the two chapters combined, so as to present a complete and continuous article.

Dr. Morland begins with a brief account of the anatomy of the urinary organs, which is followed by cases illustrative of difficulties in diagnosis, arising from their malformation, or mal-position. Then follows a short chapter, hardly two pages in length, on the diagnosis of affections of the supra-renal capsules. Here especially, the error in arrangement is apparent; for we first have but the merest skeleton of an article on diagnosis, a matter in which we are almost entirely in the dark, which would have been much more interesting, if combined with the article in Part Second, where we have an account of Addison's disease, with the researches of Harley, Séquard, Phillipaux, and others, into the function of the supra-renal capsules.

We now come to the diseases of the kidneys. Here, the author adopts, with certain modifications, the admirable arrangement of Dr. George Johnson, viz:

*Nephritis.*

A. Acute Desquamative Nephritis.

B. Chronic Desquamative Nephritis.

*Waxy Degeneration of the Kidney.*

*Non-Desquamative Disease of the Kidney.*

*Suppurative Nephritis.*

*Nephritis from Retention of Urine.*

*Pyelitis.*

*Nephritis from Renal Calculi.*

*Tubercular, or Scrofulous Disease of the Kidney.*

*Cancer of the Kidney.*

*Hæmaturia.*

The space devoted to the diagnosis of these diseases is about forty-five pages, when the author passes to the diagnosis of diseases of the ureters, bladder, and urethra. We have not the space to follow these chapters; sufficient to say, that the author gives a full account of the symptoms of these diseases, and the points which would render their diagnosis difficult.

The space occupied in the consideration of diagnosis is not quite one-fourth of the entire work; the remainder is devoted to pathology and treatment. Here we have laid down the most approved methods of treatment; and so far, it is good; but we conceive that the author has made an omission in not giving at least a brief account of the anatomy of the parts. It is impossible for the student to understand all the pathological conditions of many diseases of the kidney, without the having a clear idea of its minute anatomy. It is true that he might be expected to know this, and if he does not, it would be easy for him to find it in other works; but this is a compendium, and it is intended to contain an abstract of what is important in the diseases of the urinary organs. Such an addition would have increased the size of the work but little, and would have added much to its value.

We have made the foregoing observations, at the risk of being thought hypercritical; but in a work which has not original pretensions, and is intended to serve as a hand-book to the practitioner, convenient arrangement is of the last importance; and we hope that that will be taken into consideration if another edition be issued. On the whole, we regard it as a useful and reliable work. The illustrations we recognize; they are also reliable, if we can judge by the faithful manner in which they have gone the rounds of works on urinary diseases.

We have also an appendix, which is composed of cases illustrative of the diseases treated of in the body of the work, and articles which have been published since the text was prepared. This is by no means the least valuable part of the book, as many of the cases are very interesting and instructive, and it contains abstracts of some extremely valuable papers on urinary diseases. The work has been issued in handsome style by Blanchard & Lea, making an octavo of 560 pages.

